# The acceptability of wearable technology for long-term respiratory disease: A cross-sectional survey

**DOI:** 10.1016/j.heliyon.2024.e35474

**Published:** 2024-08-05

**Authors:** Amar J. Shah, Anita Saigal, Malik A. Althobiani, John R. Hurst, Swapna Mandal

**Affiliations:** a UCL Respiratory, University College London, UK; b Royal Free Hospital NHS Foundation Trust, London, UK; c King Abdulaziz University, Department of Respiratory Therapy, Faculty of Medical Rehabilitation Sciences, Jeddah, Makkah, Saudi Arabia

**Keywords:** Wearable technology, Chronic respiratory disease, Cross-sectional survey, Acceptability

## Abstract

Few studies have investigated the acceptability of wearable technology in patients with long-term respiratory disease. We conducted a 24-item cross-sectional survey (September 2022–February 2023), developed using four common themes universal to previously described models of technology acceptance and social behavioural therapy, to explore the acceptability of wearable technology spanning the breadth of chronic respiratory disease. A total of 74 valid survey responses were analysed with 50 % aged 51–70years; 72 % female; 63 % white British ethnicity; 79 % having an income less than £50,000, and 93 % having at least obstructive airways disease. A third of participants current used wearables with 85 % using smart watches. Most of these participants used wearables to monitor their symptoms (69 %) and as a general health measurement device (85 %). Likert scale questions (ranked 1–7) showed that participants valued accuracy and approval of wearables by regulatory bodies (median (IQR) rank score 7 (Huberty et al., 2015; Preusse et al., 2016) 6–76–7 and felt that wearables would increase their confidence in managing their long-term health condition (median (IQR) rank score 6 (Huberty et al., 2015; Preusse et al., 2016) 6–76–7. Favourable product characteristics for wearables were accuracy (73 %), easy to learn (63 %) and easy to use (50 %). They were less concerned about aesthetics (23 %) and battery life (27 %). This survey will guide future developers to produce a wearable for a population with chronic respiratory disease which will improve acceptability, usability and longevity.

## Graphical abstract

**Figure:**
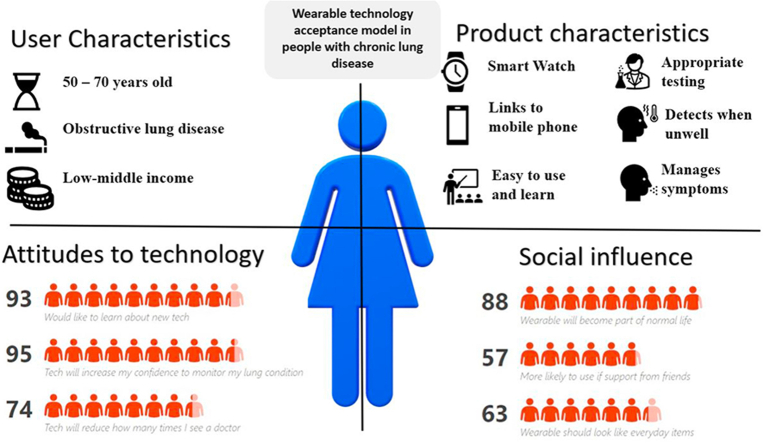

## 1 Introduction

Chronic respiratory disease affects one in five adults in the United Kingdom (UK) and is responsible for more than 700,000 hospital admissions and six-million inpatient bed-stays annually [1]. This has a significant impact on patient morbidity and the healthcare economy. Wearable technology, defined succinctly as a ‘miniature embedded computing system worn by people’ [2], has real potential to impact patient management by reducing hospital admissions, improving patient quality of life and their overall healthcare utilisation experience. This shift of healthcare from analogue to digital has also been recognised and encouraged by the World Health Organization [3]. In order to maximise the utility of wearables in healthcare, they have to be accepted and used by patients. Usability is a broad concept which includes acceptability and satisfaction of a device [4]. Importantly usability is also context specific, and how a specific context can influence acceptance and use has been highlighted previously as a research need [4,5].

Several studies previously have investigated usability of devices in healthy older adults or focussed on watch-based devices [[6], [7], [8], [9], [10]]. However, the needs of healthy adults are likely to differ from those with chronic disease. A recent systematic review aimed to look at the usability of wearable devices measuring physical activity in patients with chronic health conditions including chronic obstructive pulmonary disease (COPD). They looked at 37 studies and found usability was poorly reported and measured, with only 24 % of studies focusing on usability specifically. There was also poor recognition of human factors [4]. It is important to note that models of acceptability and thus usability include several factors that need sufficient exploration such as user characteristics, attitudes about technology, product characteristics and social influence. These factors impact widespread usability of the wearable device and are critical to assess [[11], [12], [13], [14]]. However, in the literature usability is often summarised with wear-time and adherence data, rather than assessing the true acceptability of the device [15].

There have only been a few studies that have specifically investigated the acceptability of wearable technology in patients with long-term respiratory disease. Prinable et al. [16] conducted an online survey (n = 134), to assess key attributes that can be used for long-term monitoring of breathing in patients with and without chronic asthma. They found that most participants would be willing to wear a device (62 %), with the wrist-watch being the preferred wearable (92.5 %). In patients with asthma, the main motivating factors in using the device were ‘having asthma’ and the ability to track breathing patterns when breathless. Cost was not a barrier to use, but participants felt the battery life of the device had to be at least 24-h. Simmich et al. [17], used semi-structured interviews (n = 19) in older patients with COPD. They concluded that wearables can be useful to facilitate goal setting and visualise long-term improvements. Finally, Keogh et al. [18] investigated the acceptability of a waist worn device in COPD patients (n = 25). Semi-structured interviews showed that the device was easy to use and comfortable, but due to the lack of interaction with the participant, was not considered a useful device.

There is clearly a paucity of data surrounding the different facets of acceptability in patients with chronic respiratory disease. Previous work has mainly focused on participants with obstructive airways disease and not other disease groups. Moreover, no study has broken down technology attitudes or looked at the social influence on acceptability in the UK. Therefore, the aim of this study was to explore the acceptability of wearable technology spanning the breadth of chronic respiratory disease, to enable us to start to build an acceptability model for this patient population to guide future wearable design. Secondary aims included identifying patient design preferences for wearables and identification of the impact of social norm perspectives on wearables.

## 2 Methods

This cross-sectional survey received ethical approval from the Health Research Authority and Care Research Wales (HCRA), Research Ethics Committee reference 22/NS/0017. The survey was designed to evaluate key factors impacting acceptability, based on four common themes universal to previously well described models of technology acceptance and social behavioural theory. The four common themes included:

1. Diffusion of innovation theory. The key component of this theory is that innovation ‘diffuses’ through five different categories (innovators, early adopters, early majority, late majority and laggards). The key components affecting diffusion include the innovation itself (e.g., the ability to fulfil a goal), the adopter themselves and the social system (i.e., the perception or ideology of the social pressure to adopt a particular innovation [11,19].
2. The theory of reasoned action (TRA) first described by Fishbein & Ajzen in 1975. Central to this theory is that most important determinant of a specific behavious is the intention behind the behaviour. This in turn is determined by attitudes towards the behaviour and subjective norms. The attitudes are impacted by an individuals normal beliefs and their beliefs about the outcomes of a behaviour [13].
3. The theory of planned behaviour (TPB) was developed as extension to TRA. While the TRA assumes a behaviour can be controlled voluntarily, this is not always the case. TPB introduces the concept of perceived behavioural control and refers to an individual’s perception of how easy or difficult the behaviour will be [20,21].
4. Technology acceptance model (TAM) was introduced by Fred Davis in 1989. This is based on two main factors determining acceptance. Perceived usefulness and perceived ease of use [12].

These models led to the development of four common themes of technology acceptance, echoed by Sun et al. who conducted a survey on the acceptance of personal health devices in China:

1. User characteristics: demographics, technology experience and educational background
2. Attitudes about technology: perceived usefulness, willingness to learn and privacy and trust
3. Product characteristics: ease of use, cost, accuracy and aesthetics
4. Social/wider population influence: social norm, peer pressure and social support.

An initial pool of 27 questions was developed based around the above four themes. These were subsequently reviewed by an Asthma-UK/British Lung Foundation expert patient panel to understand the suitability of the questions in terms of meeting the overall research aim and the length of the survey. Following feedback and revision of the initial 27 questions, a final survey of 24 questions was developed (Supplementary material). Questions related to the four themes as follows:

1. User characteristics – Q2, Q3, Q4, Q5, Q6, Q7, Q21, Q22, Q23, Q24
2. Attitudes about technology – Q8, Q9, Q10, Q12
3. Product characteristics – Q11, Q13, Q14, Q15, Q16,
4. Social/wide population influence – Q17, Q18, Q19

A 7-point Likert scale was used for ten questions, with scores ranging from 1: strongly disagree to 7: strongly agree. Two questions were presented in reverse order, to ensure validity, and any participant who gave inconsistent answers in either reverse question was eliminated from the valid analysis sample set.

The finalised survey was completely anonymous and was sent out to patients via respiratory clinics at a London teaching hospital, via the Asthma-UK/BLF network and through social media posts from September 2022 to February 2023. The survey responses were received either electronically (via SurveyMonkey®) or identical paper versions were provided where asked for.

Statistical analysis used both quantitative and descriptive methodology. Any survey responses with a lack of consent or no information were excluded, and any survey responses with inconsistent answers in either reverse question were also excluded. Ten questions had a 7-point Likert scale response, but one of these was dependent on prior wearable use. The internal validity of the remaining nine questions was assessed using Cronbach’s alpha. The Likert questions were summated by both median (IQR) rank scores and by percentage of positive scores. Scores of 5–7 were considered positive scores, meaning that respondents agreed with the item/question, scores of 1–3 were considered negative scores and scores of 4 were neutral values. Binomial logistic regression was used to assess whether age, gender and annual income were associated with current wearable use.

**Fig. 1:**
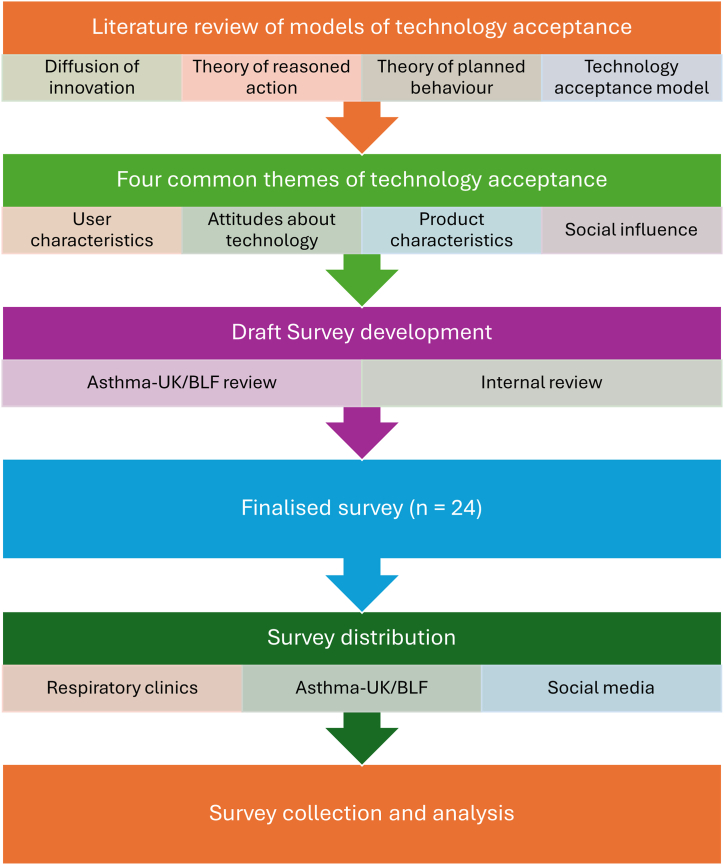
A summary of the methodology.

A summary of the methodology can be seen in Fig. 1.

## 3 Results

The survey had an initial response from 106 participants, 17 did not provide any information and 15 had inconsistent answers in the reverse questions and so were excluded. Therefore, 74 (70 %) valid survey responses were analysed (50 % aged 51–70years, 72 % female, 63 % white British ethnicity and 79 % earned less than £50,000). There was a mix of respiratory conditions (51 % asthma, 23 % chronic obstructive airways disease, 16 % sleep apnoea and 8 % interstitial lung disease), with 35 % of participants having comorbid respiratory conditions. There was no significant difference between the valid responses and those that were excluded due to inconsistent answers. This is illustrated in Table 1.

**Table 1:** Baseline demographics.

Demographic variable	Valid Response n = 74 (%)	Inconsistent answers/excluded n = 15 (%)	P value
Age			
18–21 years	0	0	0.44
22–30 years	3/72 [4]	0	
31–40 years	9/72 [13]	0	
41–50 years	8/72 [11]	1 [7]	
51–60 years	18/72 [25]	5 (33)	
61–70 years	18/72 [25]	3 [20]	
71–80 years	14/72 [19]	6 (40)	
81–90 years	0	0	
>90 years	2/72 [3]	0	
**Gender**			
Male	20/72 [28]	3 [20]	0.53
Female	52/72 (72)	12 (80)	
**Ethnicity**			
Asian other	3/71 [4]	0	0.57
Black African	1/71 [1]	0	
Black British	3/71 [4]	0	
Chinese	1/71 [1]	0	
Indian	4/71 [6]	2 [13]	
White British	45/71 (63)	12 (80)	
Mixed	3/71 [4]	1 [7]	
Other	11/71 [15]	0	
**Personal income/year**			
£0	0	0	0.42
£0–9999	9/53 [17]	1/11 [9]	
£10,000–24,999	15/53 [28]	3/11 [27]	
£25,000–49,999	18/53 (34)	2/11 [18]	
£50,000–74,999	6/53 [11]	4/11 (36)	
£75,000–99,999	1/53 [2]	0	
> £100,000	4/53 [8]	1/11 [9]	
**Chronic lung disease diagnosis (more than one answer possible)**			
COPD	17 [23]	7 (47)	0.06
Obstructive sleep apnoea	12 [16]	1 [7]	0.34
Asthma	38 (51)	8 (53)	0.89
Lung cancer	1 [1]	0	0.65
Interstitial lung disease	6 [8]	1 [7]	0.85
Bronchiectasis	14 [19]	4 [27]	0.50
Long-COVID	5 [7]	0	0.30
Other*	11 [15]	1 [7]	0.84
2-diseases	18 [24]	5 (33)	0.47
3-disease	8 [11]	1 [7]	0.63

Abbreviations: COPD = chronic obstructive pulmonary disease.

Data presented by the count of respondents and the percentage proportionate to the total count of responses for each item. Demographics were not a mandatory requirement for participants to fill in.

*included non-tuberculous mycobacteria, allergic broncho-pulmonary aspergillosis and sarcoidosis.

Overall, 26/74 (35 %) participants currently used a wearable device, with most using smart watches (58 %). None of these participants used a pedometer. Participants utilised their wearable both in relation to their lung condition and for general health purposes. With regards to their lung condition, 69 % used the wearable to monitor their symptoms, 50 % to encourage them to exercise, 15 % for medication reminders and 12 % to help predict and exacerbation. With regards to general health, 85 % used the device as a general health measurement device while 50 % used it to track their progress against general health goals. Binomial logistic regression looking at whether age (<50 years vs. >50years), gender (female vs. male), and annual income (<£50,000 vs. >£50,000) were associated with current wearable use, showed no significant differences with respective odds ratios of 0.76 (0.20–2.9), 2.31 (0.63–8.4) and 0.91 (0.21–3.9).

One Likert-scale question was dependent on whether participants had used wearable technology. For the remaining nine Likert-scale questions, Cronbach’s alpha was 0.65. The median (IQR) rank scores and percentage of positive scores are illustrated in Table 2.

**Table 2:** Median rank scores presented in descending order.

Question	Theme	Median (IQR)	Positive scores (%)
It is important the wearable technology has undergone testing in an appropriate clinical trial and has been approved by regulatory bodies.	Product characteristics	7 [6,7]	99
I would like to learn about new technology that I can wear.	Attitudes to technology	7 [6,7]	93
Wearable technology will increase my confidence to monitor my long-term lung condition at home	Attitudes to technology	6 [6,7]	95
It is important that the wearable technology links to other devices that I use to monitor my health	Product characteristics	6 [[5], [6], [7]]	85
I think wearable technology will become a normal part of everyday life in the future.	Social influence	6 [[5], [6], [7]]	88
I believe that wearable technology will reduce the number of times I see a doctor of my community team, in relation to my lung condition.	Attitudes to technology	5 [[4], [5], [6]]	74
I am more likely to use wearable technology if I have the support from my friends and family.	Social influence	5 [[4], [5], [6]]	57
The wearable technology should look the same as other everyday items so that other people don’t know I am wearing it	Social influence	5 [[4], [5], [6]]	63
I think that the wearable technology that is currently available is accurate	Attitudes to technology	5 [4,5]	51

Most participants (99 %) agreed that it was important that wearables had undergone appropriate testing in a clinical trial but only 51 % agreed that currently available wearable technology is accurate.

Several questions asked participants about their ideal product characteristics. Most participants wanted wearable technology that detected when they were becoming unwell (81 %), to help them manage their symptoms (78 %), improve their sleep (55 %) and encourage exercise (49 %). Participants were also asked to identify three characteristics of a future wearable device that would be most important to them (Question 13). Most participants (44/76) incorrectly answered this question by either choosing more or less than three characteristics. From the valid 30 responses, the commonest characteristics were delivery of accurate results (73 %), being easy to learn (63 %) and ease of use (50 %). Participants cared less about battery life (27 %), price and brand (27 %) and aesthetics (23 %).

In terms of accessing information from wearables, 76 % preferred accessing the information from their own mobile device, while 55 % wanted to access the information through the wearable itself. Only 4 % did not have any access to their information.

## 4 Discussion

This survey on wearable technology in patients with chronic respiratory disease had a 70 % completion rate. The participant demographic was of a typical age distribution seen in chronic lung disease clinics with 50 % of participants being between 50 and 70years old. Most participants were female (72 %) and from a white British ethnic background and earnt less than £50,000. While there was a good spread of different respiratory diseases, the majority had obstructive airways disease. No significant differences were seen between the valid responses and those that gave inconsistent answers.

Approximately one third of the respondents currently used a wearable device, giving an insight into potential user characteristics. This is similar to previously published data by Chandrasekaran et al. (n = 4451), who found that 30 % of adults in the United States use a wearable health device [22]. In this survey, smart watches and Fitbit devices were the commonly used devices, with most participants using the device to monitor their symptoms. This is similar to prior work Prinable et al. [16] who specifically looked at the role of wearables in an asthma population. Interestingly, only about 50 % of these patients agreed that they found the device useful, while 19 % disagreed or remained neutral. This may suggest that currently available wearables are not specific enough for patients to realise the benefit. While the previous survey by Chandrasekaran et al. found that older adults (>50 years) were less likely to use wearables compared to 18–34 years (OR 0.46–0.57), our data found no difference. This is perhaps not surprising as most respondents in our survey were above 50, and our overall sample size was small. It is important to note that other studies have echoed the results by Chandrasekaran et al. with a ‘digital gap’ still evident. For example, in the United States, 25 % of adults above 65years are not online and nearly 40 % do not own a smartphone [23]. This highlights the need to find and address barriers in the older population. Our study primarily had an older demographic and therefore will help further understand the specific needs of this older population who have chronic lung disease.

Cronbach’s alpha for the Likert-scale questions was acceptable at 0.65, suggesting adequate internal validity of the questions. Participants ranked the importance of wearables undergoing testing and approval by regulatory bodies the highest, with 99 % agreeing with this item (median rank score 7). This is important given that most wearables currently have not undergone rigorous testing, and therefore lack understanding of reliability and accuracy [14]. Some participants seemed to be aware of this, as only 51 % (median rank score 5) agreed that currently available technology is accurate. This was similar to findings by Sun et al. who also found that their respondents expressed negative attitutdes towards accuracy of peronsalised health devices [14]. Moreover, most participants listed accuracy as one of their key characteristics in future wearables, further underlining the importance of this.

**Fig. 2:**
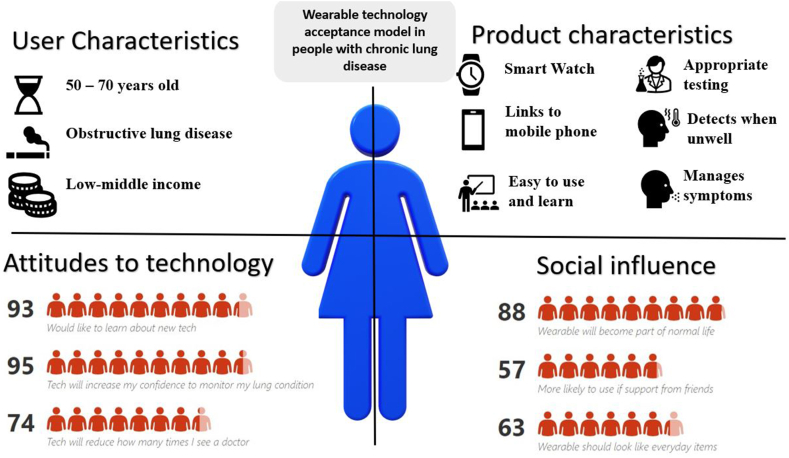
Infographic illustrating a technology acceptance model in people with chronic respiratory disease, based around four central themes.

This survey was based around four different themes, common and central to previously described models of technology acceptance. The graphical abstract and Fig. 2 illustrates a potential acceptability model for wearable technology in chronic lung disease based on these themes which shows the typical future user, their product preferences, attitudes to the technology and potential social influences.

User characteristics are important when developing new technologies. This survey showed that the average user is between 50 and 70years of age, on a low-middle income salary, is female and has obstructive airways disease. Some caution in these characteristics is advised as prior work described above has shown age can be a barrier. Increased number of female responses in this survey, may suggest women are more likely to use novel technology or simply that more women agreed to participate in the survey, leading to an element of bias. However, the large population survey by Chandrasekaran et al. [22] also found women were more likely to use wearable technology. It is also worth noting that the design of wearables and their characteristics may differ depending on gender, and future designs should take this into account [24].

Participants attitudes towards technology are vital. A population that is technology averse, will not be receptive to new technology, meaning reduced behavioural intention, fewer early innovators and less ‘diffusion’ within the target population [19]. However, our survey has shown that most participants are agreeable to learning about new technology (median rank score 7), felt that technology will increase their confidence to monitor their condition (median rank score 6) and felt that wearables would reduce the number of times they see their doctor (median rank score 5). This attitude is promising as it suggests that the behavioural intent is there.

Product characteristics are important when developing devices to help longevity and perceived usefulness. Most participants wanted wearable devices to detect when they become unwell and to help manage their symptoms. Favoured product characteristics from this survey (ease of use, mobile phone device and accuracy tested by appropriate testing), are similar to those concluded by Sun et al. [14] Interestingly this survey demonstrated characteristics such as aesthetics and data privacy were less concerning. This may come from a lack of understanding of the need to protect one’s health information. Cilliers et al. [25] investigated the privacy issues to which users are exposed to when using wearables and found that 50 % of respondents did not understand the need to protect health information and there was a lack of awareness about security issues. To ensure patients are protected by future wearables, data privacy along with the aforementioned characteristics should be maintained to ensure patient safety.

Social norms and peer pressure impact the ‘diffusion’ and acceptance of novel technology. Most participants in this survey agreed that wearables will become a normal part of everyday life in the future (median rank 6), fewer agreed that social support would increase their use. This contrasts with Sun et al. [14], who surveyed a Chinese population, where social norms were very important. This highlights important population differences and suggests novel technology should be aware of a populations' social dynamics.

There are some limitations to this survey. Firstly, the number of Likert-scale questions were too small to enable exploratory factor analysis. While the initial survey had a larger number of questions, the expert panel at Asthma-UK/BLF had felt that the length of the survey was too long. Second, the survey did not have many open/free-text questions enabling qualitative analysis which may have allowed better expression of participants’ views on wearables. Third, the majority of respondents were of White British ethnicity, meaning the views of ethnic minority communities were not represented. Moreover, prior work has shown that wearable devices are not as widely used in the minority populations [26]. This information and their views are thus vital to build a complete acceptable model for wearable technology. Their views are important not only has their social construct may differ, but also as wearables may provide incorrect readings for ethnic minority groups [27]. Future studies need to account for this. Fourth the survey had an overall small number of respondents and a complete response rate of 70 %. Although it is worth noting that in the literature a response rate of 60 % is considered acceptable [28].

In conclusion, this survey has highlighted that people living with chronic lung diseases are open to the idea of wearable devices in the future that are accurate, easy to use, easy to learn and approved by regulatory bodies. A survey focusing specifically on participants with any chronic lung disease in the UK has to our knowledge not been conducted before, making this data novel. While there was no direct question about cost, most participants of this age group had low-middle income earnings, meaning any new technology should be affordable, and focus on helping patients manage their symptoms and detect when they become unwell. Participants prioritised functionality over aesthetics. This survey will help future developers of wearable technology to produce a device in this patient population, thereby increasing the chance of patient acceptance and thus usefulness.

## Authorship statement

AJS and SM designed the survey and were responsible for the survey methodology. AJS collected the data for the survey and was involved in the initial analysis of the survey results. AS and MA contributed to further analysis. AJS and SM wrote the initial manuscript and all authors contributed to the revision and the final approval of the manuscript.

## Data availability statement

None of this data associated with this study has been deposited into a publicly available repository. However, data will be made available upon request.

## Funding

Nil.

## Ethical approval

This cross-sectional survey received ethical approval from the Health Research Authority and Care Research Wales (HCRA), Research Ethics Committee reference 22/NS/0017.

## CRediT authorship contribution statement

**Amar J. Shah:** Writing – review & editing, Writing – original draft, Validation, Software, Resources, Project administration, Methodology, Investigation, Formal analysis, Data curation, Conceptualization. **Anita Saigal:** Writing – review & editing, Methodology, Formal analysis. **Malik A. Althobiani:** Writing – review & editing, Visualization, Formal analysis, Data curation. **John R. Hurst:** Writing – review & editing, Visualization, Supervision, Project administration, Methodology. **Swapna Mandal:** Writing – review & editing, Visualization, Supervision, Methodology, Investigation, Formal analysis, Data curation, Conceptualization.

## Declaration of competing interest

The authors declare that they have no known competing financial interests or personal relationships that could have appeared to influence the work reported in this paper.
