# Supplementary material for: The acceptability of wearable technology for long-term respiratory disease: A cross-sectional survey
Source: Heliyon. 2024 Aug 5;10(16):e35474. doi: 10.1016/j.heliyon.2024.e35474 (PMC11363846; doi:10.1016/j.heliyon.2024.e35474)
Supplement: Multimedia component 1 [file mmc1.pdf]

The acceptability of wearable technology for long-term respiratory disease: a cross-sectional survey

You are being invited to participate in a research study titled: **the acceptability of wearable technology for long-term respiratory disease: a cross-sectional survey.** This study is being done by Dr Amar Shah and Dr Swapna Mandal who are respiratory doctors at the Royal Free Hospital in Hampstead, London and will form part of Dr Amar Shah's PhD thesis being done at University College London.

What is the purpose of this survey?
The purpose of this research study is to try and find out whether wearable technology would be acceptable to patients who suffer with long-term respiratory disease, for example COPD, asthma, lung fibrosis, lung cancer, bronchiectasis etc.

What is wearable technology?
Wearable technology is any electronic device that is worn by someone close to and/or on the surface of the skin. It can collect information about that person for example, body signals such as heart rate or breathing rate or oxygen levels, activity levels, sleep patterns etc. It can track these signals, monitor progress, and let that person know how the signals have changed (feedback). Most of the wearable technology that is available to buy has not been tested in a research study and the results can sometimes be inaccurate. Examples of wearable technology can be seen in the picture below:

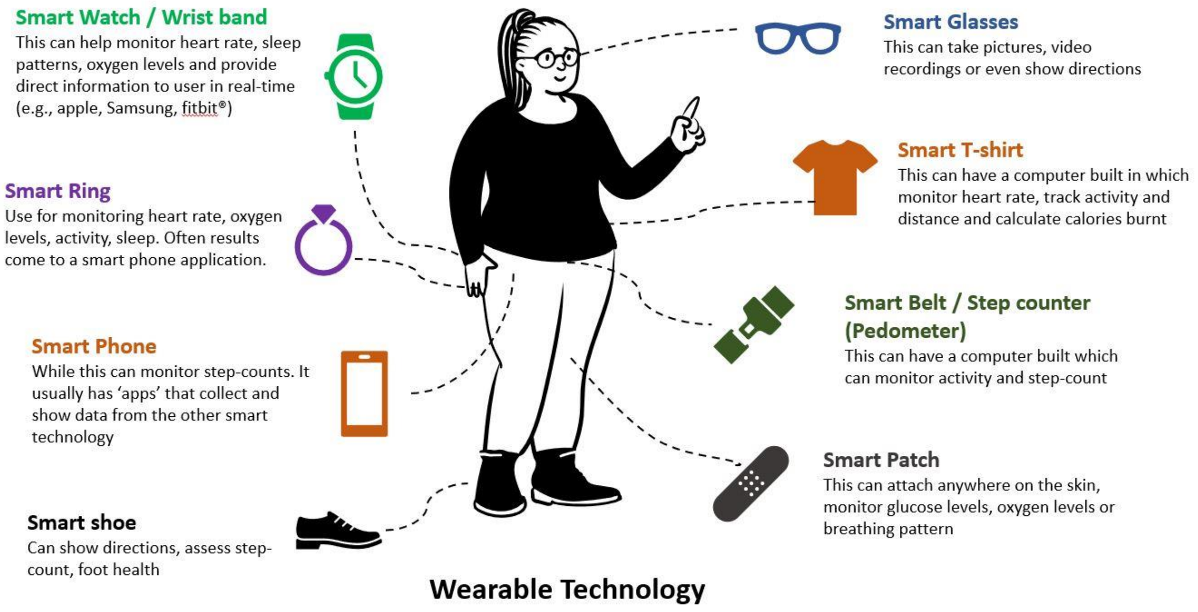

We are interested in getting patients' views on the following:

1) Is new wearable technology something they would be interested in trying in the future?
2) Do they think new wearable technology could help them with their lung conditions?
3) What features are important in any new technology?

What will the information be used for?
The information from this survey will be used to hopefully guide future development of new technology that is specific to patients with respiratory disease. It will also give us an idea on what features are important to patients when designing new technology. We will aim to publish the information in a medical journal. Your answers are completely anonymous and you will not be identifiable from the information.

How will the information be processed and stored?
All the information on this survey is treated confidentially by SurveyMonkey. All data is collected and the content is stored in a manner consistent with industry security standards in accordance with the Data Protection Act 2018 and is General Data Protection Regulation (GDPR) compliant. All the information we collect about you during the course of the research will be kept strictly confidential and only the research team directly involved in the study will have access to the answers. You will not be able to be identified in any publications or reports from this research. We will share the anonymised data with selected non-commercial third parties, such as respiratory charities.

We believe there are no known risks associated with this research study. However, as with any online related activity the risk of a breach is always possible.

Your participation in this study is entirely voluntary and will take approximately 15-20 minutes (including reading the information sheet) to complete. You do not have to answer any questions you do not want to. If you are happy to take part you will be asked to fill a consent form.

For further information about the study please download the participant information sheet: [https://drive.google.com/file/d/1dIxP0LmC20Uxe7-WerHDIuWgVEnAiy_8/view?usp=sharing] and retain this for your records.

1. I have read the above information and the patient information sheet. Please tick one of the following options:

☐ I wish to proceed with this study and am happy to give my consent

☐ I do not wish to proceed with this study

2. Which of the following lung conditions are you currently affected by? If not listed please use the other box and write the condition down. (Please tick all that apply).

☐ Chronic obstructive lung disease (COPD)

☐ Obstructive sleep apnoea (OSA)

☐ Asthma

☐ Lung cancer

☐ Interstitial lung disease (ILD), including idiopathic pulmonary fibrosis (IPF) and sarcoidosis

☐ Bronchiectasis

☐ Long COVID

☐ Other ………………………………………………………………..

3. Do you currently wear something to monitor your health and wellbeing?

☐ Yes (Go to Q4) ☐ No (Go to Q8, page 5)

4. What type of device do you currently wear to monitor your health and wellbeing? Please choose the type of device from the following list. If not listed please use the 'other' box and write down the name of the device. (Please tick all that apply).

☐ Smart watch (e.g., Apple watch, Samsung watch, Garmin watch or others that are designed to monitor your health and well-being)

☐ Fitbit®

☐ Smart glasses that are designed to monitor your health and wellbeing

☐ Smart Ring (e.g., oura rings or others that monitor your health and wellbeing)

☐ Pedometer or step counter

☐ Clothing (including T-shirt, vest etc./ that monitor your health and wellbeing with sensors)

☐ Patch (e.g., glucose monitoring patch, or others that monitor your health and wellbeing)

☐ Belts (e.g., across your chest or tummy or elsewhere that are able to monitor your health and wellbeing)

☐ Other (please specify) …………………………………………………………………………………………………

5. What do you use this wearable technology for, specifically in relation to your lung condition? (Tick all that apply)

☐ To monitor symptoms of my lung condition through measurements, such as breathing rate, respiratory effort, oxygen levels etc.

☐ To remind me to take medication for my lung condition

☐ To encourage exercise or other self-care for my lung condition

☐ To help predict when I might become unwell from my lung condition

☐ Not applicable

☐ Other (please specify) …………………………………………………………………………………………………….

6. What do you use this wearable technology for, in relation to your general health? (Tick all that apply)

☐ General health measurements e.g., heart rate, sleep quality, oxygen levels

☐ To track my progress against general health goals

☐ To remind me to take medication

☐ To encourage exercise or other self-care

☐ Not applicable

☐ Other (please specify) ………………………………………………………………………………………………………

7. I find the device I use to monitor my health useful.

Strongly disagree	Disagree	Somewhat disagree	Neutral	Somewhat Agree	Agree	Strongly Agree
☐	☐	☐	☐	☐	☐	☐

8. I would like to learn about new technology that I can wear.

Strongly disagree	Disagree	Somewhat disagree	Neutral	Somewhat Agree	Agree	Strongly Agree
☐	☐	☐	☐	☐	☐	☐

9. Wearable technology will increase my confidence to monitor my long-term lung condition at home.

Strongly disagree	Disagree	Somewhat disagree	Neutral	Somewhat Agree	Agree	Strongly Agree
☐	☐	☐	☐	☐	☐	☐

10. I believe that wearable technology will reduce the number of times I see a doctor or my community, in relation to my lung condition.

Strongly disagree	Disagree	Somewhat disagree	Neutral	Somewhat Agree	Agree	Strongly Agree
☐	☐	☐	☐	☐	☐	☐

11. Wearable technology that helps me with the following would be useful for me. (Tick all that apply)

☐ Detects when I am becoming unwell

☐ Helps me to manage my symptoms (e.g., breathlessness, cough, chest tightness etc.)

☐ Reminds me to take my medication

☐ Encourages me to exercise and become more active

☐ Improves my sleep quality

☐ Other (please specify) …………………………………………………………………………………………….

12. I think that the wearable technology that is currently available is accurate.

Strongly disagree	Disagree	Somewhat disagree	Neutral	Somewhat Agree	Agree	Strongly Agree
☐	☐	☐	☐	☐	☐	☐

13. Which of the following 3 characteristics of wearable technology are most important to you?

☐ Easy to learn

☐ Easy to use

☐ Battery life

☐ Price and brand

☐ Look and feel (aesthetics)

☐ Accurate (correct) results

☐ Privacy of data collected

☐ Other (please specify) ………………………………………………………………………………………………………

14. How would you prefer to access the information recorded / monitored from the wearable technology (Tick all that apply)

☐ Own mobile phone

☐ Through the wearable technology itself (e.g., screen directly on technology)

☐ A computer or tablet

☐ An extra monitor that attaches to the wearable device

☐ I would not want to access any of the information and would prefer it to go directly to my healthcare provider (e.g., doctor, nurse, physiotherapist)

☐ Other (please specify) ………………………………………………………………………………………………………..

15. It is important that the wearable technology links to other devices that I use to monitor my health (e.g., peak flow meter, exercise diary, symptom diary).

Strongly disagree	Disagree	Somewhat disagree	Neutral	Somewhat Agree	Agree	Strongly Agree
☐	☐	☐	☐	☐	☐	☐

16. It is important that the wearable technology has undergone testing in an appropriate clinical trial and has been approved by regulatory bodies.

Strongly disagree	Disagree	Somewhat disagree	Neutral	Somewhat Agree	Agree	Strongly Agree
☐	☐	☐	☐	☐	☐	☐

17. The wearable technology should look the same as other everyday items so that other people don't know I am wearing it.

Strongly agree	Agree	Somewhat Agree	Neutral	Somewhat Disagree	Disagree	Strongly Disagree
☐	☐	☐	☐	☐	☐	☐

18. I think wearable technology will become a normal part of everyday life in the future.

Strongly disagree	Disagree	Somewhat disagree	Neutral	Somewhat Agree	Agree	Strongly Agree
☐	☐	☐	☐	☐	☐	☐

19. I am more likely to use wearable technology if I have the support from my friends and family

Strongly disagree	Disagree	Somewhat disagree	Neutral	Somewhat Agree	Agree	Strongly Agree
☐	☐	☐	☐	☐	☐	☐

20. Do you have any other comments or thought on wearable technology to manage your lung condition?

Demographic data

The next part of the survey is to give us a bit of information about you. This information is completely anonymous but feel free to end the survey if you do not wish to answer this section.

21. Please select what age range you fit into.

☐ 18 – 21 years
☐ 22 – 30 years
☐ 31 – 40 years
☐ 41 – 50 years
☐ 51 – 60 years
☐ 61 – 70 years
☐ 71 – 80 years
☐ 81 – 90 years
☐ Above 90 years

22. What is your gender?

☐ Male
☐ Female
☐ Non-binary
☐ Intersex
☐ Transgender
☐ Other …………………………………………..
☐ Prefer not to say

23. What is your ethnicity?

☐ Asian Other
☐ Bangladeshi
☐ Black African
☐ Black British
☐ Black Caribbean
☐ Chinese
☐ Hispanic or Latino
☐ Indian
☐ Pakistani
☐ White British
☐ Mixed
☐ Other (please specify)

24. Which of the following best describes your approximate household income last year?

☐ £0
☐ Between £1 and £9,999
☐ Between £10,000 and £24,999
☐ Between £25,000 and £49,999
☐ Between £50,000 and £74,999
☐ Between £75,000 and £99,999
☐ Over £100,000
☐ Prefer not to answer
